# Unsupervised Phoneme and Word Discovery From Multiple Speakers Using Double Articulation Analyzer and Neural Network With Parametric Bias

**DOI:** 10.3389/frobt.2019.00092

**Published:** 2019-10-01

**Authors:** Ryo Nakashima, Ryo Ozaki, Tadahiro Taniguchi

**Affiliations:** Emergent Systems Laboratory, College of Information Science and Engineering, Ritsumeikan University, Shiga, Japan

**Keywords:** word discovery, phoneme discovery, parametric bias, Bayesian model, neural network

## Abstract

This paper describes a new unsupervised machine-learning method for simultaneous phoneme and word discovery from multiple speakers. Phoneme and word discovery from multiple speakers is a more challenging problem than that from one speaker, because the speech signals from different speakers exhibit different acoustic features. The existing method, a nonparametric Bayesian double articulation analyzer (NPB-DAA) with deep sparse autoencoder (DSAE) only performed phoneme and word discovery from a single speaker. Extending NPB-DAA with DSAE to a multi-speaker scenario is, therefore, the research problem of this paper.This paper proposes the employment of a DSAE with parametric bias in the hidden layer (DSAE-PBHL) as a feature extractor for unsupervised phoneme and word discovery. DSAE-PBHL is designed to subtract speaker-dependent acoustic features and speaker-independent features by introducing parametric bias input to the DSAE hidden layer. An experiment demonstrated that DSAE-PBHL could subtract distributed representations of acoustic signals, enabling extraction based on the types of phonemes rather than the speakers. Another experiment demonstrated that a combination of NPB-DAA and DSAE-PBHL outperformed other available methods accomplishing phoneme and word discovery tasks involving speech signals with Japanese vowel sequences from multiple speakers.

## 1. Introduction

Infants discover phonemes and words from speech signals uttered by their parents and the individuals surrounding them (Saffran et al., 1996a,b). This process is performed without transcribed data (i.e., labeled data) in a manner that differs from most of the recent automatic speech recognition (ASR) systems. In the field of developmental robotics, a robot is regarded as the model of a human infant. Developing a machine-learning method that enables a robot to discover phonemes and words from unlabeled speech signals is crucial (Cangelosi and Schlesinger, 2015). This study aims to create a machine-learning method that can discover phonemes and words from unlabeled data for developing a constructive model of language acquisition similar to human infants and to leverage the large amount of unlabeled data spoken by multiple speakers in the context of developmental robotics (Taniguchi et al., 2016a). The main research question of this paper is how to extend an existing unsupervised phoneme and word discovery method [i.e., nonparametric Bayesian double articulation analyzer (NPB-DAA) with a deep sparse autoencoder (DSAE)] and develop a method that can achieve unsupervised phoneme and word discovery from multiple speakers.

Most available ASR systems are trained using transcribed data that must be prepared separately from the learning process (Kawahara et al., 2000; Dahl et al., 2012; Sugiura et al., 2015). By using certain supervised learning methods and model architectures, an ASR can be developed with a very large transcribed speech data corpus (i.e., a set of pairs of text and acoustic data). However, human infants are capable of discovering phonemes and words through their natural developmental process. They do not need transcribed data. Moreover, they discover phonemes and words at a time when they have not developed the capability to read text data. This evidence implies that infants discover phonemes and words in an unsupervised manner via sensor–motor information.

It is widely established that 8-month-old children can infer chunks of phonemes from the distribution of acoustic signals (Saffran et al., 1996b). Caregivers generally utter a sequence of words rather than an isolated word in their infant-directed speech (Aslin et al., 1995). Therefore, word segmentation and discovery is essential for language acquisition. Saffran et al. explained that human infants use three types of cues for word segmentation: prosodic, distributional, and co-occurrence (Saffran et al., 1996a,b). Prosodic cues include information related to prosody, such as intonation, tone, stress, and rhythm. Distributional cues include transitional probabilities between sounds and appearance frequencies of a certain sequence of sounds. Co-occurrence cues relate sounds and entities in the environment. For example, a child may notice that “dog” is often uttered in the presence of a pet.

In this study, we focus on distributional cues. Saffran et al. also reported that 8-month-old infants could perform word segmentation from continuous speech using solely distributional cues (Saffran et al., 1996a). Thiessen et al. reported that distributional cues appeared to be used by human infants by the age of 7 months (Thiessen and Saffran, 2003). This is earlier than for other cues. However, the computational models that discover phonemes and words from human speech signals have not been completely explored in the fields of developmental robotics and natural language or speech processing (Lee and Glass, 2012; Lee et al., 2013, 2015; Kamper et al., 2015; Taniguchi et al., 2016b,c). The unsupervised word segmentation problem has been studied for a long time (Brent, 1999; Venkataraman, 2001; Goldwater et al., 2006, 2009; Johnson and Goldwater, 2009; Mochihashi et al., 2009; Sakti et al., 2011; Magistry, 2012; Chen et al., 2014; Takeda and Komatani, 2017). However, their models did not assume the existence of phoneme recognition errors, Therefore, if they are applied to phoneme sequences recognized by a phoneme recognizer, which usually involves a lot of phoneme recognition errors, their performance significantly deteriorates. Neubig et al. extended the sampling procedure proposed by Mochihashi to handle word lattices that could be obtained from an ASR system (Neubig et al., 2012). However, the improvement was limited, and they did not consider phoneme acquisition. It was indicated that feedback information from segmented words was essential to phonetic category acquisition (Feldman et al., 2013). Subsequent to these studies, several others were conducted to develop unsupervised phoneme and word discovery techniques (Kamper et al., 2015; Lee et al., 2015; Taniguchi et al., 2016b,c). This type of research is very similar to the development of unsupervised learning of speech recognition systems, which transforms speech signals into sequences of words. The development of an unsupervised machine-learning method that can discover words and phonemes is important for providing fresh insight into developmental studies from a computational perspective. In this study, we employ NPB-DAA (Taniguchi et al., 2016b).

The double articulation structure in spoken language is a characteristic structural feature of human language (Chandler, 2002). When we develop an unsupervised machine-learning method based on probabilistic generative models (i.e., the Bayesian approach), it is critical to clarify our assumption about the latent structure embedded in observation data. The double articulation structure is a two-layer hierarchical structure. A sentence is generated by stochastic transitions between words, a word corresponds to a deterministic sequence of phonemes, and a phoneme exhibits similar acoustic features. This double articulation structure is universal for languages.

Taniguchi et al. (2016b) developed NPB-DAA to enable a robot to obtain knowledge of phonemes and words in an unsupervised manner, even if the robot did not know the number of phonemes and words, a lists of phonemes, or words and transcriptions of the speech signals. Taniguchi et al. introduced the DSAE to improve the performance of NPB-DAA. They demonstrated that it outperformed a conventional off-the-shelf ASR system trained using transcribed data (Taniguchi et al., 2016c). The main research purpose of developing NPB-DAA with DSAE was to develop an unsupervised phoneme and word-discovery system that could be regarded as a computational explanation of the process of human language acquisition, rather than to develop a high-performance ASR system.

The experiments conducted in (Taniguchi et al., 2016b,c) used speech data obtained from only one speaker. The NPB-DAA with DSAE did not assume learning environments where a robot learned phonemes and words from multiple speakers. The direct application of NPB-DAA with DSAE to a multi-speaker scenario is highly likely to be ineffective. Extending NPB-DAA with DSAE to a multi-speaker scenario is, therefore, the research objective here.

**Figure 1:**
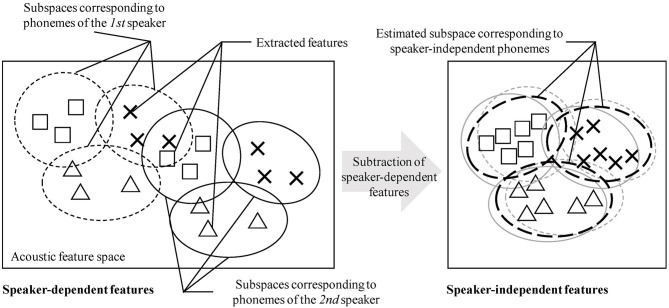
Schematic of speaker-dependent and speaker-independent acoustic features. Each shape represents each phoneme (e.g., “a,” “i,” and “u”), and each type of circle represents each speaker. In the left feature space, each phoneme is embedded in a speaker-dependent manner. If a clustering method is performed on this feature space, speaker-dependent phonemes will be discovered. In the speaker-independent feature space shown in the right, no phoneme depends on each speaker. If speaker-independent feature representations are obtained by subtracting speaker-dependent features, an appropriate clustering method is expected to achieved phoneme discovery in an unsupervised manner.

In the studies of unsupervised phoneme and word discovery, learning from speech signals obtained from multiple speakers has been recognized as challenging (Dunbar et al., 2017; Kamper et al., 2017). To explain the essential challenge, an example of the discrimination of “a” from “i” is considered. Figure 1 provides a schematic of the explanation that follows. Fundamentally, the phoneme discovery problem can be regarded as a type of clustering problem. A machine-learning method for unsupervised phoneme and word discovery should be capable of identifying and distinguishing clusters of “a” and “i.” If the acoustic feature distributions of “a” and “i” are sufficiently different, a proper unsupervised machine-learning method could form two clusters (i.e., acoustic categories). For example, DSAE can form reasonable feature representations, and NPB-DAA can simultaneously categorize phonemes and words. If explicit feature representations are formed, a standard clustering method (e.g., Gaussian mixture model) can also perform phoneme discovery to a certain extent. However, in a multi-speaker setting, the acoustic feature distribution of each phoneme can differ, depending on the speakers. That is, “a” from the first speaker and “a” from the second speaker will exhibit different feature distributions in the feature space. The direct application of a clustering method on the data tends to form different clusters (i.e., phoneme categories) for “a” from the first and second speakers. To enable a robot to acquire phonemes and words from the speech signals obtained from multiple speakers, it must omit, cancel, or subtract speaker-dependent information from the observed speech signals. In Figure 1, the speaker-dependent features and the speaker-independent features are extracted. If speaker-independent feature representations can be formed similarly, the proposed clustering method (e.g., NPB-DAA) will likely identify phonemes from the extracted features.

How to omit, cancel, or subtract speaker-dependent information is a crucial challenge in unsupervised phoneme and word discovery from multiple speakers. Conventional studies on ASR, which can use transcribed data, adopt an approach that omits the differences between multiple speakers by using transcribed data. Although “a” from speakers A and B exhibit different distributions, by using label data, the pattern recognition system can learn that both distributions should be mapped to label “a.” In the scenario of supervised learning, deep learning-based speech recognition systems adopt these types of approaches by exploiting a considerable amount of labeled data and the flexibility of neural networks (Hannun et al., 2014; Amodei et al., 2016; Chan et al., 2016; Chiu et al., 2018). This approach was not suitable for this study, because the research question is different. With this study, we intend to investigate unsupervised phoneme and word discovery. The system should not use transcription. Instead, we focus on speaker index information (i.e., “who is speaking now?”) to subtract speaker-dependent acoustic features. We assume that the system can sense “who is speaking now?” (i.e., speaker index)^1^. To apply the speaker index and subtract speaker-dependent information from acoustic features, we employed the concept of parametric bias in the study of neural networks. Neural networks have been demonstrated to exhibit rich representation learning capability and has been widely used for more than a decade (Hinton and Salakhutdinov, 2006; Bengio, 2009; Le et al., 2011; Krizhevsky et al., 2012; Liu et al., 2014). In the context of developmental robotics, Tani and Ogata et al. proposed and explored recurrent neural networks with parametric bias (Tani et al., 2004; Ogata et al., 2007; Yokoya et al., 2007). Parametric bias is an additional input that can function as a *gray* switch to modify the function of the neural network. In our study, the speaker index was manually provided as an input of parametric bias as a part of dataset. Moreover, neural networks can encode independent feature information into each neuron if it is trained under suitable conditions. This is called “disentanglement.” The property of disentanglement has attracted much attention in recent studies (Bengio, 2009; Chen et al., 2016; Higgins et al., 2017). The arithmetic manipulability rooting on this characteristic of the neural network has also gained attention. It was demonstrated that Word2Vec (i.e., skip-gram for word embedding) could predict the representation vector of “Paris” by subtracting the vector of “Japan” from that of “Tokyo” and adding that of “France” (Mikolov et al., 2013a,b). Considering these concepts, we propose DSAE-PBHL to subtract speaker-dependent information.

**Figure 2:**
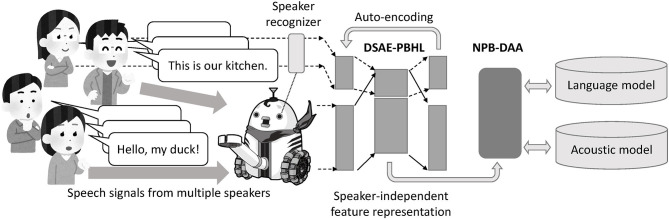
Overview of proposed method, NPB-DAA with DSAE-PBHL. First, a robot observes spoken utterances with speaker indices using a speaker recognition method (e.g., face recognition). DSAE-PBHL, which accepts speaker-dependent features and the speaker index as input, extracts speaker-independent feature representations and passes them to NPB-DAA. NPB-DAA segments the feature sequences and identifies words and phonemes (i.e., language and acoustic models) in an unsupervised manner.

The overview of our approach, unsupervised phoneme and word discovery using NPB-DAA with DSAE-PBHL, is depicted in Figure 2. First, a robot observes spoken utterances with speaker indices using a speaker recognition method (e.g., face recognition). DSAE-PBHL, which accepts speaker-dependent features and speaker index as input, extracts speaker-independent feature representations and passes them to NPB-DAA. NPB-DAA then segments the feature sequences and identifies words and phonemes (i.e., language and acoustic models) in an unsupervised manner.

We propose an unsupervised learning method that can identify words and phonemes directly from speech signals uttered by multiple speakers. The method based on NPB-DAA and DSAE-PBHL is a form of unsupervised learning, except for the use of an index of a speaker, which is assumed to be estimated by the robot (i.e., a model of a human infant).

The remainder of this paper is organized as follows: Section 2 describes existing methods to create a background for this study. Section 3 briefly describes the proposed method: a combination of NPB-DAA and DSAE-PBHL. Section 4 describes two experiments that evaluate the effectiveness of the proposed method using actual sequential Japanese vowel speech signals. Section 5 concludes this paper.

## 2. Background

The proposed method comprises NPB-DAA and DSAE-PBHL, an extension of DSAE (see Figure 2). In this section, we briefly introduce NPB-DAA (Taniguchi et al., 2016b). Then, we describe DSAE (Ng, 2011; Liu et al., 2015; Taniguchi et al., 2016c).

### 2.1. NPB-DAA

**Figure 3:**
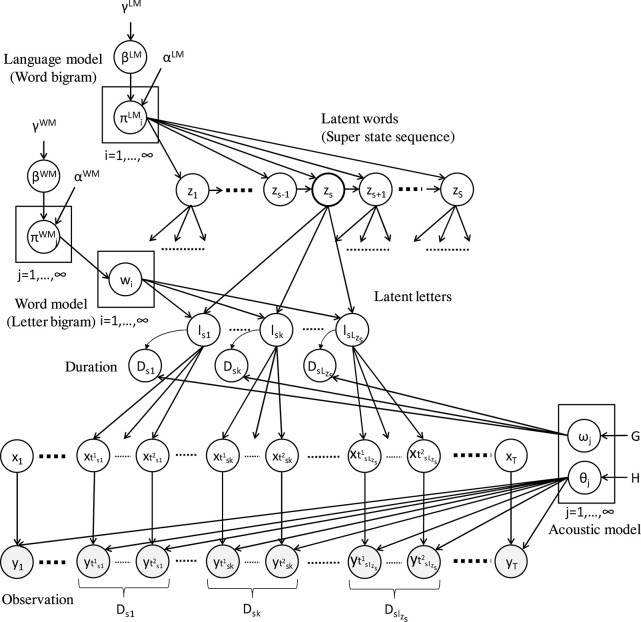
Graphical model of HDP-HLM (Taniguchi et al., 2016b). HDP-HLM has language, word, and acoustic models as latent variables of an integrated probabilistic generative model. HDP-HLM can infer these models and latent sequences of words (i.e., latent words) and phonemes (i.e., latent letters) using a blocked Gibbs sampler.

The hierarchical Dirichlet process hidden language model (HDP-HLM) is a probabilistic generative model that models double articulation structures (i.e., two-layer hierarchy) characteristic of spoken human language (Taniguchi et al., 2016b). Mathematically, HDP-HLM is a natural extension of the HDP hidden semi-Markov model (HDP-HSMM), which is a type of generalization of the hidden Markov model (HMM) (Johnson and Willsky, 2013). NPB-DAA is the name of an unsupervised learning method for phoneme and word discovery based on HDP-HLM. Figure 3 shows the graphical model of HDP-HLM.

Whereas HDP-HMM assumes that the latent variable transits between them following Markov process, HDP-HLM assumes that the latent variable, the index of phoneme, transits according to the word bigram language model. In HDP-HSMM, a superstate persists for a certain duration determined by the duration distribution and outputs an observation using a corresponding emission distribution. Meanwhile, in HDP-HLM, a latent word persists for a certain duration, and the model outputs observations with a sequential transition of latent letters (i.e., phonemes). Note that, in the HDP-HLM terminology, the variable corresponding to a phoneme is called a “latent letter.” The variable corresponding to a word is called a “latent word.”

Because HMM-based ASR has language and acoustic models, HDP-HLM has both these as latent variables in its generative model. Because of the nature of Bayesian non-parametrics (i.e., Dirichlet process prior), HDP-HLM can determine the number of phonemes and words via the inference process. It is not necessary to fix the number of phonemes and words (i.e., the number of latent letters and words) beforehand. In the graphical model, the *s*-th latent word corresponds to superstate *z*_*s*_. Superstate *z*_*s*_ = *i* has a sequence of latent letters, *w*_*i*_ = (*w*_*i*1_, …, *w*_*ik*_, …, *w*_*iL*_*i*__). Here, *w*_*ik*_ is the index of the *k*-th latent letter of the *i*-th latent word. *L*_*i*_ represents the string length of *w*_*i*_. The generative process of HDP-HLM is as follows:

(1) $$\begin{array}{r} \beta^{LM}\sim \text{GEM}\left(\gamma^{LM}\right) \end{array}$$

(2) $$\begin{array}{r} \pi_{i}^{LM}\sim \text{DP}\left(\alpha^{LM},\beta^{LM}\right)\;i=1,2,\ldots ,\infty \end{array}$$

(3) $$\begin{array}{r} \beta^{WM}\sim \text{GEM}\left(\gamma^{WM}\right) \end{array}$$

(4) $$\begin{array}{r} \pi_{j}^{WM}\sim \text{DP}\left(\alpha^{WM},\beta^{WM}\right)\;j=1,2,\ldots ,\infty \end{array}$$

(5) $$\begin{array}{r} w_{ik}\sim \pi_{w_{ik-1}}^{WM}\;i=1,2,\ldots ,\infty \;k=1,2,\ldots ,L_{i} \end{array}$$

(6) $$\begin{array}{r} \left(\theta_{j},\omega_{j}\right)\sim H\times G\;j=1,2,\ldots ,\infty \end{array}$$

(7) $$\begin{array}{r} z_{s}\sim \pi_{z_{s-1}}^{LM}\;s=1,2,\ldots ,S \end{array}$$

(8) $$\begin{array}{r} l_{sk}\sim w_{z_{s}k}\;s=1,2,\ldots ,S\;k=1,2,\ldots ,L_{z_{s}} \end{array}$$

(9) $$\begin{array}{r} D_{sk}\sim g\left(\omega_{l_{sk}}\right)\;s=1,2,\ldots ,S\;k=1,2,\ldots ,L_{z_{s}} \end{array}$$

(10) $$\begin{array}{l} x_{t}=l_{sk}\;t=t_{sk}^{1},\ldots ,t_{sk}^{2}\\ \;t_{sk}^{1}=\underset{s^{\prime }<s}{\sum }D_{s^{\prime }}+\underset{k^{\prime }<k}{\sum }D_{sk^{\prime }}+1\;t_{sk}^{2}=t_{sk}^{1}+D_{sk}-1 \end{array}$$

(11) $$\begin{array}{r} y_{t}\sim h\left(\theta_{x_{t}}\right)\;t=1,2,\ldots ,T \end{array}$$

Here, GEM represents a stick-breaking process (SBP), and DP represents the Dirichlet process (DP). β^*WM*^ represents the based measure of the Dirichlet process for the word model, and α^*WM*^ and γ^*WM*^ are hyperparameters of DP and SBP, respectively. A word model is a prior distribution of a sequence of latent letters composing a latent word. DP(α^*WM*^, β^*WM*^) generates a transition probability, $\pi_{j}^{WM}$, which is a categorical distribution over the subsequent latent letter of the *j*-th latent letter. Similarly, β^*LM*^, DP(α^*LM*^, and β^*LM*^) represent the based measure of the DP for the language model and hyperparameters of DP and SBP, respectively. DP(α^*LM*^, β^*LM*^) generates a transition probability, $\pi_{i}^{LM}$, which is a categorical distribution over the subsequent latent letter of the *i*-th latent letter. The notations, *LM* and *WM*, represent language and word models, respectively. The emission distribution, *h*, and duration distribution, *g*, have parameters θ_*j*_ and ω_*j*_ drawn from the base measures, *H* and *G*, respectively. The variable, *z*_*s*_, is the *s*-th word in the latent word sequence. Moreover, *D*_*s*_ is the duration of *z*_*s*_, *l*_*sk*_ = *w*_*z*_*s*_*k*_ is the *k*-th latent letter of the *s*-th latent word, and *D*_*sk*_ is its duration. Variables, *y*_*t*_ and *x*_*t*_, represent the observation and latent state corresponding to a latent letter at time *t*. The times, $t_{sk}^{1}$ and $t_{sk}^{2}$, represent the start and end times, respectively, of *l*_*sk*_.

If we assume the duration distribution of a latent letter to follow a Poisson distribution, the model exhibits an effective mathematical feature because of the reproductive property of Poisson distributions. The duration, *D*_*sk*_, is drawn from *g*(ω_*l*_*sk*__). Therefore, the duration of *w*_*z*_*s*__ is $D_{s}=\overset{L_{z_{s}}}{\underset{k=1}{\sum }}D_{sk}$. If we assume *D*_*sk*_ to follow a Poisson distribution (i.e., *g* is a Poisson distribution), *D*_*s*_ also follows a Poisson distribution. In this case, the parameter of the Poisson duration distribution of *w*_*z*_*s*__ becomes $\overset{L_{z_{s}}}{\underset{k=1}{\sum }}\omega_{l_{sk}}$. The observation, *y*_*t*_, corresponding to *x*_*t*_ = *l*_*s*(*t*)*k*(*t*)_, is generated from *h*(θ_*x*_*t*__). Here, *s*(*t*) and *k*(*t*) are mappings that indicate the corresponding word, *s*, and the letter, *k*, at time *t*.

Following the process described above, HDP-HLM can generate time-series data exhibiting a latent double articulation structure. In this study, we assumed that the observation, *y*_*t*_, corresponded to the acoustic features. In summary, {ω_*j*_, θ_*j*_}_*j* = 1, 2, …, ∞_ represents acoustic models, and ${\left\{\pi_{i}^{LM},w_{i}\right\}}_{i=1,2,\ldots ,\infty }$ represents language models. The inference of the latent variables of this generative model corresponds to the simultaneous discovery of phonemes and words. An inference procedure for HDP-HLM was proposed in Taniguchi et al. (2016b), based on the blocked Gibbs sampler for HDP-HSMM proposed by Johnson and Willsky (2013). The pseudocode of the procedure is described in Algorithm 1. In this paper, we omit the details of the procedure. For further details, please refer to the original paper (Taniguchi et al., 2016b).

**Algorithm 1:** Blocked Gibbs sampler for HDP-HLM (Taniguchi et al., 2016b).

Initialize all parameters.
Observe *M* time series data, ${\left\{y_{1:T_{m}}^{m}\right\}}_{m\in \left\{1,2,\ldots ,M\right\}}$.
**repeat**
**for** *m* = 1 to *M* **do**
// Backward-filtering procedure
**for** *i* = 1 to *N* **do**
*B*_*T*_*m*__(*i*)←1
**end for**
**for** *t* = *T*_*m*_ − 1 to 0 **do**
**for** *i* = 1 to *N* **do**
$B_{t}\left(i\right)=\overset{N}{\underset{j=1}{\sum }}B_{t}^{*}\left(j\right)p\left(z_{s\left(t+1\right)}=j|z_{s\left(t\right)}=i\right)$
$B_{t}^{*}\left(i\right)=\overset{T_{m}-t}{\underset{d=1}{\sum }}B_{t+d}\left(i\right)p\left(D_{s\left(t+1\right)}=d|z_{s\left(t+1\right)}=i\right)$
*p*(*y*_*t*+1:*t*+*d*_|*i, d*)
**end for**
**end for**
// Forward-sampling procedure
*s* ← 1, $D_{s}^{\text{sum}}\leftarrow 0$
**while** $D_{s}^{\text{sum}}<T_{m}$ **do**
// Sampling a superstate representing a latent word
$z_{s}^{m}\sim p\left(z_{s}^{m}|y_{1:T_{m}}^{m},z_{s-1}^{m},F_{D_{s}^{\text{sum}}}=1\right)$
// Sampling duration of the superstate
$D_{s}^{m}\sim p\left(D_{s}^{m}|z_{s},F_{D_{s}^{\text{sum}}}=1\right)$
$D_{s+1}^{\text{sum}}\leftarrow D_{s}^{\text{sum}}+D_{s}^{m}$
*s* ← *s* + 1
**end while**
*S*^*m*^ ← *s* − 1
**for** *s* = 1 to *S*_*m*_ **do**
// Sampling a tentative latent letter sequence
${\bar{w}}_{s}^{m}\sim P\left(w|y_{D_{s-1}^{\text{sum}}+1:D_{s}^{\text{sum}}}^{m},{\left\{\pi_{j}^{WM},\omega_{j},\theta_{j}\right\}}_{j=1,2,\ldots ,J}\right)$
**end for**
**end for**
// Update model parameters
**for** *j* = 1 to *J* **do**
$\left\{\omega_{j},\theta_{j}\right\}\sim P\left(\omega_{j},\theta_{j}|{\left\{z_{1:S_{m}}^{m},D_{1:S_{m}}^{m},{\bar{w}}_{1:S_{m}}^{m},y_{1:T_{m}}^{m}\right\}}_{m}\right)$
**end for**
${\left\{\pi_{i}^{LM}\right\}}_{i},\beta^{LM}\sim P\left({\left\{\pi_{i}^{LM}\right\}}_{i},\beta^{LM}|{\left\{z_{1:S_{m}}^{m}\right\}}_{m}\right)$
**for** *i* = 1 to *N* **do**
$w_{i}\sim p\left(w_{i}|{\left\{z_{1:S_{m}}^{m},D_{1:S_{m}}^{m},y_{1:T_{m}}^{m}\right\}}_{m}\right)$
**end for**
${\left\{\pi_{i}^{WM}\right\}}_{i},\beta^{WM}\sim p\left({\left\{\pi_{i}^{WM}\right\}}_{i},\beta^{WM}|{\left\{w_{i}\right\}}_{i}\right)$
**until** a predetermined exit condition is satisfied.

### 2.2. DSAE

In Taniguchi et al. (2016c), features extracted using DSAE were used as the input of NPB-DAA. DSAE is a representation learning method comprising several sparse autoencoders (SAE) (Ng, 2011). By stacking several autoencoders and assigning penalty terms to the loss function for improving robustness and sparsity, DSAE is obtained. In DSAE, each SAE attempts to minimize the reconstruction errors and learn efficient and essential representations of the input data (i.e., speech signals).

**Figure 4:**
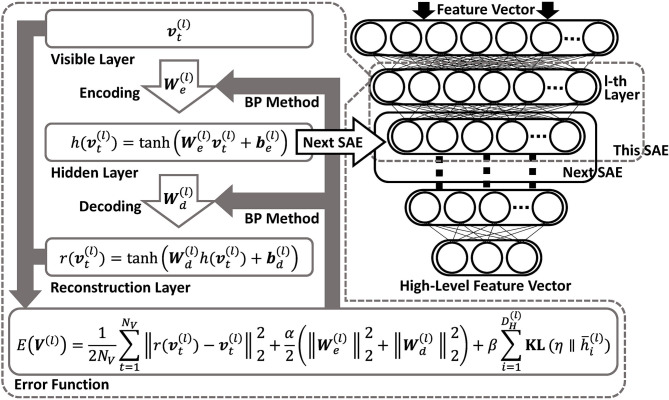
Overview of DSAE. DSAE comprises stacked autoencoders having regularization terms in their objective functions to improve generalization capability. Each layer is trained to minimize reconstruction errors.

Figure 4 shows an overview of DSAE. In this study, we assumed that the original input of speech signals were converted into Mel frequency cepstral coefficients (MFCC), following the process described in Taniguchi et al. (2016c). The time-series data is obtained as a matrix, $\text{O}\in {\mathbb{R}}^{D_{O}\times N_{O}}$. Here, *N*_*O*_ represents the amount of data. The acoustic feature at time *t* is represented by ${\text{o}}_{t}\in {\mathbb{R}}^{D_{O}}$, as follows:

(12) $$\begin{array}{r} {\text{o}}_{t}={\left(o_{t,1},o_{t,2},\ldots ,o_{t,D_{O}}\right)}^{T}, \end{array}$$

where *D*_*O*_ represents the dimension of vector **o**_*t*_.

In this study, the hyperbolic tangent function, **tanh**(·), was used as the activation function of SAE. To fit the input data to the range of **tanh**(·) for reconstruction, the input vector **o**_*t*_ was normalized as follows:

(13) $$v_{t}={\left(v_{t,1},v_{t,2},\ldots ,v_{t,D_{O}}\right)}^{T}\;v_{t,d}=2\left(\frac{o_{t,d}-O_{\text{min},d}}{O_{\text{max},d}-O_{\text{min},d}}\right)-1,$$

where *O*_max,*d*_ and *O*_min,*d*_ are the maximum and minimum values, respectively, of the *d*-th dimension of all data: *o* ∈ *O*.

Each SAE has an encoder and a decoder. The encoder of the *l*-th SAE in DSAE is

(14) $$\begin{array}{r} {\text{h}}_{t}^{\left(l\right)}=\text{tanh}\left({\text{W}}_{e}^{\left(l\right)}{\text{v}}_{t}^{\left(l\right)}+{\text{b}}_{e}^{\left(l\right)}\right). \end{array}$$

Following this function, regarding the *t*-th datum, a vector of the *l*-th layer, ${\text{v}}_{t}^{\left(l\right)}$, is transformed to a vector of the *l*-th hidden layer, ${\text{h}}_{t}^{\left(l\right)}\in {\mathbb{R}}^{D_{H}^{\left(l\right)}}$. Each decoder is represented as follows: the vector of the *l*-th layer, ${\text{r}}_{t}^{\left(l\right)}\in {\mathbb{R}}^{D_{V}^{\left(l\right)}}$, is obtained from the vector of the *l*-th reconstruction layer.

(15) $$\begin{array}{r} {\text{r}}_{t}^{\left(l\right)}=\text{tanh}\left({\text{W}}_{d}^{\left(l\right)}\text{h}\left({\text{v}}_{t}^{\left(l\right)}\right)+{\text{b}}_{d}^{\left(l\right)}\right), \end{array}$$

where ${\text{W}}_{e}^{\left(l\right)}\in {\mathbb{R}}^{D_{H}^{\left(l\right)}\times D_{V}^{\left(l\right)}}$ in (14) is the weight matrix, and ${\text{b}}_{e}^{\left(l\right)}\in {\mathbb{R}}^{D_{H}^{\left(l\right)}}$ is the bias of the encoder. Moreover, ${\mathbb{R}}^{D_{V}^{\left(l\right)}}$ and ${\mathbb{R}}^{D_{H}^{\left(l\right)}}$ represent the dimensions of the input and hidden layers, respectively. Similarly,${\text{W}}_{d}^{\left(l\right)}\in {\mathbb{R}}^{D_{V}^{\left(l\right)}\times D_{H}^{\left(l\right)}}$ in (15) is the weight matrix of the decoder, and ${\text{b}}_{d}^{\left(l\right)}\in {\mathbb{R}}^{D_{V}^{\left(l\right)}}$ is the bias.

The loss function is defined as follows:

(16) $$\begin{array}{l} E\left({\text{V}}^{\left(l\right)}\right)=\frac{1}{2N_{V}}\overset{N_{V}}{\underset{t=1}{\sum }}||{\text{r}}_{t}^{\left(l\right)}-{\text{v}}_{t}^{\left(l\right)}{||}_{2}^{2}+\frac{\alpha }{2}\left(||{\text{W}}_{e}^{\left(l\right)}{||}_{2}^{2}+||{\text{W}}_{d}^{\left(l\right)}{||}_{2}^{2}\right)\\ \;+\beta \overset{D_{H}^{\left(l\right)}}{\underset{i=1}{\sum }}\text{KL}\left(\eta ||{\bar{h}}_{i}^{\left(l\right)}\right). \end{array}$$

Because the dimensions of the weight matrices, ${\text{W}}_{e}^{\left(l\right)}$ and ${\text{W}}_{d}^{\left(l\right)}$, were high, it was necessary to prevent the penalty terms, ${\text{W}}_{e}^{\left(l\right)}$, ${\text{W}}_{d}^{\left(l\right)}$ (i.e., L2 norm), and $\beta \overset{D_{H}^{\left(l\right)}}{\underset{i=1}{\sum }}\text{KL}\left(\eta ||{\bar{h}}_{i}^{\left(l\right)}\right)$ (i.e., sparse term). This is the Kullback–Leibler divergence between the two Bernoulli distributions having η and ${\bar{h}}_{i}^{\left(l\right)}$ as their parameters. This type of DSAE is introduced in Ng (2011). The following are details of the sparse term:

(17) $$\begin{array}{l} KL(\eta ||{\bar{h}}_{i}^{\left(l\right)})=\eta \log\frac{\eta }{{\bar{h}}_{i}^{\left(l\right)}}+(1-\eta )\log\frac{1-\eta }{1-{\bar{h}}_{i}^{\left(l\right)}}\;\\ \;{\bar{h}}_{i}^{\left(l\right)}=\frac{1}{2}(1+\frac{1}{N_{V}}\sum_{t=1}^{N_{V}}h_{t,i}^{\left(l\right)}), \end{array}$$

where η ∈ ℝ is a parameter that regulates sparsity. Moreover, ${\bar{h}}_{i}^{\left(l\right)}$ represents the average of the *i*-th dimension's activation. The vector, ${\bar{\text{h}}}^{\left(l\right)}\in {\mathbb{R}}^{D_{H}^{\left(l\right)}}$ ${\bar{h}}_{i}^{\left(l\right)}$, is defined by combining ${\bar{h}}_{i}^{\left(l\right)}$. In this study, to calculate the sparse term, ${\bar{\text{h}}}^{\left(l\right)}$ was normalized from (−1, 1) to (0, 1), because **tanh**(·) was used as an activation function. To optimize the DSAE, a simple back-propagation method was used (Rumelhart et al., 1985).

As described above, we can obtain the weight matrices, $H^{\left(l\right)}=\left(h_{1}^{\left(l\right)},...,h_{t}^{\left(l\right)}\right)\in {\mathbb{R}}^{D_{H}^{\left(l\right)}\times N_{V}}$, for obtaining ${\text{V}}^{\left(l+1\right)}\in {\mathbb{R}}^{D_{H}^{\left(l\right)}\times N_{V}}$. By stacking the optimized SAE's, high-level feature representations can be obtained.

## 3. DSAE-PBHL

This section describes our proposed DSAE-PBHL, which employs a feature extractor that extracts speaker-independent features from multiple speakers. We use DSAE-PBHL with NPB-DAA for unsupervised phoneme and word discovery in a multi-speaker scenario.

**Figure 5:**
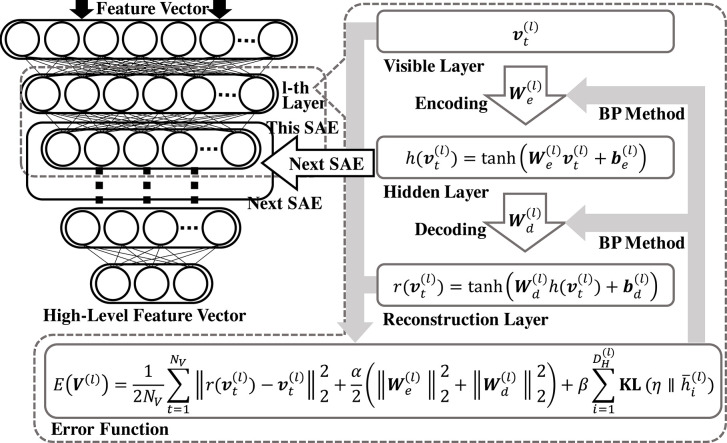
Overview of DSAE-PBHL. DSAE-PBHL has parametric bias input only for a part of the hidden layer. Neurons in the hidden layer receiving projections from parametric-bias neurons are encouraged to encode speaker-dependent information that can predict the speaker index. On the contrary, the other neurons in the hidden layer are expected to be discouraged to encode speaker-dependent information and code speaker-independent information.

This section describes DSAE-PBHL, which subtracts speaker-dependent features in the latent space. DSAE-PBHL is a DSAE with a final layer. A part of this layer receives speaker index information from the other network. The layer is used to subtract speaker-dependent information in a self-organizing manner. Figure 5 shows an overview of DSAE-PBHL. The *L*-th layer (i.e., the final layer) receives parametric bias input from a different network (see the right nodes of the network in Figure 5). However, the vital aspect of DSAE-PBHL is that some of the nodes in the final layer receives a projection from the network representing speaker index information. The input vector, ${\text{v}}_{t}^{\left(L\right)}\in {\mathbb{R}}^{D_{V}^{\left(L\right)}}$, comprises the parametric bias, ${\text{p}}_{t}^{\left(L\right)}\in {\mathbb{R}}^{D_{P}^{\left(L\right)}}$, and a vector, ${\text{x}}_{t}^{\left(L\right)}\in {\mathbb{R}}^{D_{X}^{\left(L\right)}}$, obtained from the (*L* − 1)-th SAE.

(18) $$\begin{array}{r} {\text{v}}_{t}^{\left(L\right)}={\left({\text{x}}_{t}^{\left(L\right)},{\text{p}}_{t}^{\left(L\right)}\right)}^{T}\in {\mathbb{R}}^{D_{V}^{\left(L\right)}}, \end{array}$$

where $D_{X}^{\left(L\right)}$ and $D_{P}^{\left(L\right)}$ represent the dimensions of ${\text{x}}_{t}^{\left(L\right)}$ and ${\text{p}}_{t}^{\left(L\right)}$, respectively. Note that $D_{V}^{\left(L\right)}=D_{X}^{\left(L\right)}+D_{P}^{\left(L\right)}$.

Next, the vector of the *L*-th hidden layer, ${\text{h}}_{t}^{\left(L\right)}\in {\mathbb{R}}^{D_{H}^{\left(L\right)}}$, ${\text{x}}_{t}^{\left(L\right)}$,${\text{p}}_{t}^{\left(L\right)}$, is defined using ${\text{z}}_{t}^{\left(L\right)}\in {\mathbb{R}}^{D_{Z}^{\left(L\right)}}$,${\text{s}}_{t}^{\left(L\right)}\in {\mathbb{R}}^{D_{S}^{\left(L\right)}}$ as follows:

(19) $$\begin{array}{r} {\text{h}}_{t}^{\left(L\right)}={\left({\text{z}}_{t}^{\left(L\right)},{\text{s}}_{t}^{\left(L\right)}\right)}^{T}\in {\mathbb{R}}^{D_{H}^{\left(L\right)}}, \end{array}$$

where $D_{Z}^{\left(L\right)}$ and $D_{S}^{\left(L\right)}$ represent the dimensions of ${\text{z}}_{t}^{\left(L\right)}$ and ${\text{s}}_{t}^{\left(L\right)}$, respectively. Note that $D_{H}^{\left(L\right)}=D_{Z}^{\left(L\right)}+D_{S}^{\left(L\right)}$.

The encoder of the *L*-th SAE used (14) in a similar fashion as the general DSAE. However, the weight matrix of the encoder was trained to map the input vectors, ${\text{x}}_{t}^{\left(L\right)}$ and ${\text{p}}_{t}^{\left(L\right)}$, to the latent vectors, ${\text{z}}_{t}^{\left(L\right)}$ and ${\text{s}}_{t}^{\left(L\right)}$, in the hidden layer and generate speaker-independent feature representations and speaker-identifiable representations.

(20) $$\begin{array}{r} {\text{W}}_{e}^{\left(L\right)}=\left(\begin{array}{cc} {\text{W}}_{\text{z},\text{x}}^{\left(L\right)} & {\text{W}}_{\text{z},\text{p}}^{\left(L\right)}\\ {\text{W}}_{\text{s},\text{x}}^{\left(L\right)} & {\text{W}}_{\text{s},\text{p}}^{\left(L\right)} \end{array}\right)\in {\mathbb{R}}^{D_{H}^{\left(L\right)}\times D_{V}^{\left(L\right)}}, \end{array}$$

where, ${\text{W}}_{\text{z},\text{x}}^{\left(L\right)}\in {\mathbb{R}}^{D_{z}^{\left(L\right)}\times D_{x}^{\left(L\right)}}$, ${\text{W}}_{\text{z},\text{p}}^{\left(L\right)}\in {\mathbb{R}}^{D_{z}^{\left(L\right)}\times D_{p}^{\left(L\right)}}$, ${\text{W}}_{\text{s},\text{x}}^{\left(L\right)}\in {\mathbb{R}}^{D_{s}^{\left(L\right)}\times D_{x}^{\left(L\right)}}$, ${\text{W}}_{\text{s},\text{p}}^{\left(L\right)}\in {\mathbb{R}}^{D_{s}^{\left(L\right)}\times D_{p}^{\left(L\right)}}$, ${\text{W}}_{\text{z},\text{p}}^{\left(L\right)}=0$.

Similarly, the decoder function (15) was used, and the weight matrix of the decoder function was modified as follows:

(21) $$\begin{array}{r} {\text{W}}_{d}^{\left(L\right)}=\left(\begin{array}{cc} {\text{W}}_{\text{x},\text{z}}^{\left(L\right)} & {\text{W}}_{\text{x},\text{s}}^{\left(L\right)}\\ {\text{W}}_{\text{p},\text{z}}^{\left(L\right)} & {\text{W}}_{\text{p},\text{s}}^{\left(L\right)} \end{array}\right)\in {\mathbb{R}}^{D_{V}^{\left(L\right)}\times D_{H}^{\left(L\right)}}, \end{array}$$

where ${\text{W}}_{\text{x},\text{z}}^{\left(L\right)}\in {\mathbb{R}}^{D_{x}^{\left(L\right)}\times D_{z}^{\left(L\right)}}$, ${\text{W}}_{\text{x},\text{s}}^{\left(L\right)}\in {\mathbb{R}}^{D_{x}^{\left(L\right)}\times D_{s}^{\left(L\right)}}$, ${\text{W}}_{\text{p},\text{z}}^{\left(L\right)}\in {\mathbb{R}}^{D_{p}^{\left(L\right)}\times D_{z}^{\left(L\right)}}$, ${\text{W}}_{\text{p},\text{s}}^{\left(L\right)}\in {\mathbb{R}}^{D_{p}^{\left(L\right)}\times D_{s}^{\left(L\right)}}$, and ${\text{W}}_{\text{p},\text{z}}^{\left(L\right)}=0$.

Furthermore, the error function and optimization method were identical to those of the general DSAE. After the training phase, ${\text{z}}_{t}^{\left(L\right)}$ was obtained by excluding ${\text{s}}_{t}^{\left(L\right)}$ from the vector of the *L*-th hidden layer. ${\text{h}}_{t}^{\left(L\right)}$ and was used as a feature vector (i.e., observation, of NPB-DAA). The reason we considered it likely that ${\text{z}}_{t}^{\left(L\right)}$ encoded a speaker-independent feature representation is that the network was trained to cause ${\text{s}}_{t}^{\left(L\right)}$ to have a speaker-identifiable representation. This was because ${\text{s}}_{t}^{\left(L\right)}$, alone, was forced to contribute to reconstructing the speaker-index information (i.e., parametric bias). As Figure 5 shows, ${\text{s}}_{t}^{\left(L\right)}$ was connected only to the input of the parametric bias (i.e., speaker index). If ${\text{z}}_{t}^{\left(L\right)}$ involves speaker-dependent information that can be used to predict the speaker index, the representation is redundant. Therefore, such speaker-dependent information is likely to be mapped onto ${\text{s}}_{t}^{\left(L\right)}$. Thus, it is likely that ${\text{z}}_{t}^{\left(L\right)}$ becomes encoding information that does not contribute to the speaker identification task (i.e., it becomes speaker-independent information).

## 4. Experiment

To evaluate the proposed method, we conducted two experiments. First, we tested whether DSAE-PBHL could extract speaker-independent feature representations using speech signals representing isolated Japanese vowels and an elementary clustering method. Second, we tested whether NPB-DAA with DSAE-PBHL could successfully perform unsupervised phoneme and word discovery from speech signals obtained from multiple speakers.

### 4.1. Common Conditions

In the following two experiments, we used the common dataset. The procedure of creating data was identical to that used in previous papers (Taniguchi et al., 2016b,c). We asked two male and two female Japanese speakers to read 30 artificial sentences aloud once at a natural speed, and we recorded their voice using a microphone. In total, 120 audio data items were recorded. We named the two female datasets as K-DATA and M-DATA and the two male datasets as H-DATA and N-DATA. The 30 artificial sentences were prepared using five artificial words {aioi, aue, ao, ie, uo} comprising five Japanese vowels {a, i, u, e, o}. By reordering the words, 25 two-word sentences (e.g., “ao aioi,” “uo aue,” and “aioi aioi”) and five three-word sentences (i.e., “uo aue ie,” “ie ie uo,” “aue ao ie,” “ao ie ao,” and “aioi uo ie”) were prepared. The set of two-word sentences comprised all feasible pairs of the five words (5 × 5 = 25). The set of three-word sentences were determined manually. This dataset imitated the dataset used in Taniguchi et al. (2016c), where NPB-DAA with DSAE were proposed and evaluated on a dataset using a single speaker for comparison. NPB-DAA requires huge computational cost, and unsupervised phoneme and word discovery from a large-scale dataset remains a very hard problem. Therefore, we evaluate our method on this small dataset.

The input speech signals were provided as MFCCs, which have been widely used in ASR studies. The recorded data were encoded into 39-dimensional MFCC time series data using the HMM Toolkit (HTK)^2^. The frame size and shift were set to 25 and 10 ms, respectively. 12-dimensional MFCC data were obtained as input data by eliminating the power information from the original 13-dimensional MFCC data. As a result, 12-dimensional time-series data at a frame rate of 100 Hz were obtained.

In DSAE-PBHL, 39-dimensional MFCC was compressed by DSAE, whose variation in the dimensions was 39 → 20 → 10 → 6. The speaker index was provided to the final layer as a 4-dimensional input. In the final layer, the dimensions of ${\text{z}}_{t}^{\left(L\right)}$ and ${\text{s}}_{t}^{\left(L\right)}$ were 3 and 3, respectively. We used **z**^(*L*)^ as an input of clustering methods (e.g., k-means, Gaussian mixture models (GMM), and NPB-DAA). In DSAE, the 39-dimensional MFCC was compressed by DSAE, whose variation in the dimensions was 39 → 20 → 10 → 6 → 3. The parameters in DSAE were set as α = 0.003, β = 0.7, and η = 0.5.

### 4.2. Experiment 1: Vowel Clustering Based on DSAE-PBHL

This experiment evaluated whether the DSAE-PBHL could extract speaker-independent representations from the perspective of a phoneme-clustering task rather than a word-discovery task.

#### 4.2.1. Conditions

For quantitative evaluation, we applied two elementary clustering methods (i.e., k-means and GMM) to the extracted feature vectors to examine whether the DSAE-PBHL extracted speaker-independent feature representations. If the elementary clustering methods could identify clusters corresponding to each vowel, it would imply that each phoneme formed clustered distributions to a certain extent. The clustering performance was quantified with the adjusted Rand index (ARI), which is a standard evaluation criterion of clustering. We also tested three types of coding of parametric bias (i.e., sparse coding and codings 1 and 2, Table 1). As a baseline method, we employed DSAE and MFCC. Furthermore, we applied DSAE and the clustering methods separately to the four datasets (i.e., H-DATA, K-DATA, M-DATA, and N-DATA) and calculated the average ARI. This result can be considered an upper limit of performance. The codes of scikit-learn^3^ were used for k-means and GMM. The number of clusters of methods was fixed as five (i.e., the exact number). Regarding the other hyperparameters, the default settings of scikit-learn were used. The other settings followed the common conditions described in section 4.1.

**Table 1:** ARI in the phoneme-clustering task.

**Method**	**k-means**	**GMM**	**PB: [H-PB], [K-PB], [M-PB], [N-PB]**
DSAE-PBHL (Sparse coding)	**0.536**	**0.519**	[0,0,0,1], [0,0,1,0], [0,1,0,0], [1,0,0,0]
DSAE-PBHL (coding 1)	0.514	0.429	[0,0,0,1], [0,0,1,0], [0,0,1,1], [0,1,0,0]
DSAE-PBHL (coding 2)	0.448	0.362	[0,0,1,1], [0,1,1,0], [1,1,0,0], [1,0,0,1]
DSAE	0.212	0.222	
MFCC	0.243	0.182	
Upper limit	0.626	0.599	

*The underlined values represent the highest scores within the comparative methods*.

#### 4.2.2. Results

Table 1 presents the ARI averaged over 20 trials for k-means, GMM, and each method. This result demonstrates that DSAE-PBHL exhibited significantly higher performance than DSAE and MFCC in the representation learning of acoustic features from multiple speakers in phoneme clustering. Among the three coding methods, sparse coding (i.e., one-hot vector) achieved the best score. In numerous cases of deep learning, sparse coding exhibited effective characteristics. Therefore, this result appears to have been consistent. However, even with different cases of encoding methods, DSAE-PBHL outperformed other methods. As considered likely, DSAE-PBHL did not attain the upper limit. However, it reduced the difference.

**Figure 6:**
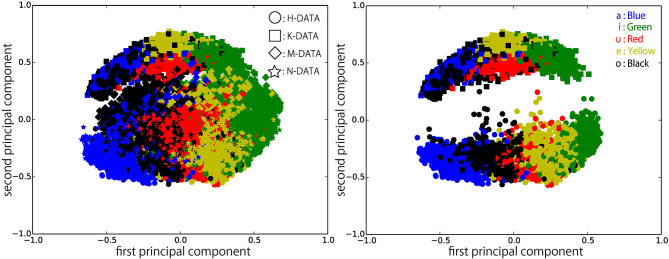
Feature representations extracted by DSAE visualized using PCA. **(Left)** all data, **(Right)** H-DATA and K-DATA.

**Figure 7:**
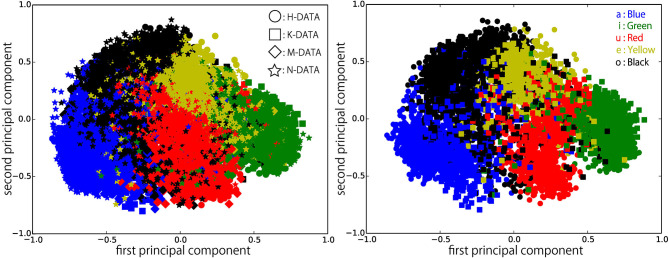
Feature representations extracted by DSAE-PBHL (sparse coding) visualized using PCA. **(Left)** all data, **(Right)** H-DATA and K-DATA.

**Figure 8:**
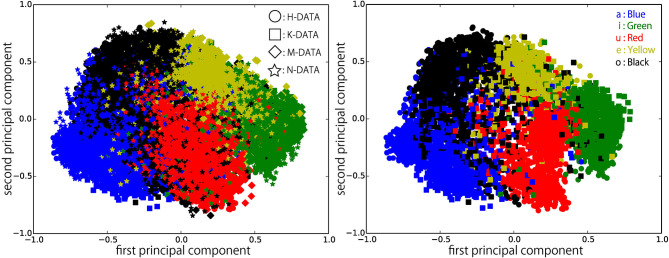
Feature representations extracted by DSAE-PBHL (Coding 1) visualized with PCA. **(Left)** all data, **(Right)** H-DATA and K-DATA.

**Figure 9:**
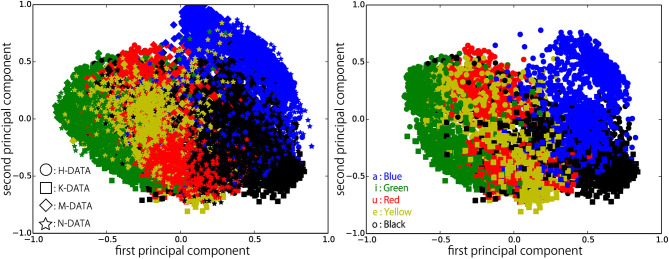
Feature representations extracted by DSAE-PBHL (Coding 2) visualized with PCA. **(Left)** all data, **(Right)** H-DATA and K-DATA.

Figures 6–9 visualize feature representations extracted by DSAE and DSAE-PBHL with three types of coding. The final 3-dimensional representation is mapped to a 2-dimensional space using principal component analysis (PCA) for the purpose of visualization. In each figure, the left side reveals a scatter plot of the data from the four speakers, and the right shows the scatter plot of the data from H-DATA and K-DATA (i.e., a male and a female speaker). On the one hand, it was observed that DSAE formed speaker-dependent distributions (see Figure 6). For example, “a” from H-DATA and “a” from K-DATA formed entirely different clusters in the feature space. On the other hand, DSAE-PBHL formed speaker-independent representations to a certain extent (Figures 7–9).

The right side of Figure 6 shows a clear split between the data from speaker H and those from speaker K. This implies that speech signals from different speakers form different clusters in the feature space. In that formed by DSAE, “o” spoken by H was more similar to “a” spoken by H than “o” spoken by K. The first principal component correlated to the type of phonemes and the second principal component correlated to the speakers. This clearly shows that DSAE formed hugely speaker-dependent feature spaces. In contrast, the two figures in Figure 7 did not show a big difference. This implies that feature representations of phonemes from every speaker and those from an individual speaker are distributed in a similar manner. Figures 8, 9 also had similar tendency. This means that DSAE-PBHL successfully formed speaker-independent feature spaces. This is quantitatively presented in Table 1.

### 4.3. Experiment 2: Simultaneous Phoneme and Word Discovery From Multiple Speakers Using NPB-DAA With DSAE-PBHL

This experiment evaluated whether NPB-DAA with DSAE-PBHL could discover phonemes and words from speech signals from multiple speakers.

#### 4.3.1. Conditions

The hyperparameters for the latent language model were set to γ^*LM*^ = 10.0 and α^*LM*^ = 10.0. The maximum number of words was set to seven for weak-limit approximation. The hyperparameters of the duration distributions were set to α = 200 and β = 10. Those of the emission distributions were set to $\mu_{0}=0,\sigma_{0}^{2}=1.0,\kappa_{0}=0.01,$ and ν_0_ = 17 = (dimension+5). The Gibbs sampling procedure was iterated 100 times for NPB-DAA. 20 trials were performed using different random-number seeds. Sparse coding of parametric bias was employed as the coding method of the speaker index. We compared NPB-DAA with DSAE-PBHL, NPB-DAA with MFCC, and NPB-DAA with DSAE. Similar to Experiment 1, we calculated the performance of NPB-DAA with DSAE, which learned speakers separately, as an upper limit of the model. Moreover, we used the off-the-shelf speech recognition system, Julius^4^, which has a pre-existing true dictionary comprising {aioi, aue, ao, ie, uo} to output ARI reference values. We used two types of Julius: the HMM-based model and the deep neural network (DNN) model: Julius DNN.

#### 4.3.2. Results

Similar to Experiment 1, Table 2 presents ARIs for each condition. The rows with “(MAP)” list the score when NPB-DAA exhibits the highest likelihood. The other rows list the average score of 20 trials. Column SS represents the single-speaker setting. Speech signals from different speakers are input separately and learned independently. This condition is considered an upper limit of the proposed model. Columns AM and LM illustrate whether the method uses pre-trained acoustic and language model (i.e., uses transcribed data), respectively. This demonstrates that NPB-DAA with DSAE-PBHL (MAP) (i.e., our proposed method) outperformed the previous models. However, it did not outperform the upper-limit method and Julius DNN. On the other hand, it is noteworthy that NPB-DAA with DSAE outperformed Julius, which was trained in a supervised manner.

**Table 2:** ARIs in phoneme- and word-discovery tasks.

**Method**	**Letter ARI**	**Word ARI**	**SS**	**AM**	**LM**
NPB-DAA with DSAE-PBHL (MAP)	**0.597**	**0.373**			
NPB-DAA with DSAE-PBHL	0.445	0.308			
NPB-DAA with DSAE (MAP)	0.160	0.073			
NPB-DAA with DSAE	0.234	0.139			
NPB-DAA with MFCC (MAP)	0.281	0.115			
NPB-DAA with MFCC	0.297	0.104			
Upper-Limit (speaker-dependence): NPB-DAA with DSAE (MAP)	**0.621**	**0.627**	✓		
Upper-Limit (speaker-dependence): NPB-DAA with DSAE	0.523	0.448	✓		
Julius (triphone + word dictionary)	0.552	0.599	–	✓	✓
Julius DNN (triphone + word dictionary)	**0.693**	**0.791**	–	✓	✓

*The underlined values represent the highest scores within the comparative methods. The bold values represent the highest scores within a class of baseline methods*.

Table 3 presents correlation coefficients between ARIs and log-likelihood for each feature extractor. A high correlation between ARI and log-likelihood indicates that the extracted features are suitable for the generative model, i.e., HDP-HLM, for clustering. DSAE-PBHL had higher correlation coefficients than the others. The result also suggests that DSAE-PBHL formed a better feature space for speech signals from multiple speakers.

**Table 3:** Correlation coefficients between letter and word ARIs and log-likelihood in phoneme- and word-discovery tasks.

**Method**	**DSAE-PBHL**	**DSAE**	**MFCC**
Letter ARI	**0.297**	0.032	0.059
Word ARI	**0.392**	–0.053	0.013

*The underlined values represent the highest scores within the comparative methods*.

This result indicates that DSAE-PBHL can reduce the adverse effect of obtaining speech signals from multiple speakers and that the simultaneous use of NPB-DAA can achieve direct phoneme and word discovery from speech signals obtained from multiple speakers, to a certain extent.

## 5. Conclusion

This paper proposed a new method, NPB-DAA with DSAE-PBHL, for direct phoneme and word discovery from multiple speakers. DSAE-PBHL was developed to reduce the negative effect of speaker-dependent acoustic features in an unsupervised manner by using a speaker index required to be obtained through another speaker recognition method. This can be regarded as a more natural computational model of phoneme and word discovery by humans, because it does not use transcription. Human infants acquire knowledge of phonemes and words from interactions with parents and other individuals that come into contact with the child. We assumed that an infant could recognize and distinguish speakers by considering certain other features (e.g., visual face recognition). This study was aimed at enabling DSAE-PBHL to subtract speaker-dependent acoustic features and extract speaker-independent features. The first experiment demonstrated that DSAE-PBHL could subtract distributed representations of acoustic signals, enabling the extraction of speaker-independent feature representations to a certain extent. The performance was quantitatively evaluated. The second experiment demonstrated that the combination of NPB-DAA and DSAE-PBHL outperformed the available unsupervised learning methods in phoneme- and word-discovery tasks with speech signals with Japanese vowel sequences from multiple speakers.

The future challenges are as follows: The experiment was performed on vowel signals. However, applying NPB-DAA to more natural speech corpora is our future challenge. It will involve consonants, which exhibit more dynamic features than vowels. However, achieving unsupervised phoneme and word discovery from natural corpora, including consonants and common vocabularies, continues to be a challenging problem. Tada et al. applied NPB-DAA with a variety of feature extraction methods (Yuki Tada, 2017). However, they obtained limited performance. Therefore, in this study, we focused on vowel data. Extending our studies to more natural spoken language is one of our intention.

Applying the method to larger corpora is another challenge. In this regard, the computational cost is high, and the method to address data from multiple speakers are problematic. We consider our proposed method to have overcome one of these barriers. Recently, Ozaki et. al. reduced the computational cost of NPB-DAA significantly (Ryo Ozaki, 2018). Therefore, we consider our contribution to be effective for further study of unsupervised phoneme and word discovery.

This paper proposed DSAE-PBHL as a proof-of-concept. DSAE-PBHL is regarded a type of conditioned neural network. Recently, the relationship between autoencoder and probabilistic generative model have been recognized via variational autoencoders (Kingma and Welling, 2013). From a broader perspective, we propose using conditioned deep generative models to obtain disentangled representations to extract speaker-independent acoustic representations. In the field of speech synthesis, voice conversion methods using a generative adversarial network have been studied (Kameoka et al., 2018). We intend to explore the relationship between our proposal and those studies and integrate them in future research.

It was demonstrated that DSAE-PBHL could mitigate the negative effects of multiple speakers by using parametric bias. However, speech signals from different speakers may depend on other attributes (e.g., recording environment). In this study, we did not distinguish recording-dependent features from speaker-dependent features, but we attempted to subtract such information by using DSAE-PBHL in an unsupervised manner. Therefore, each parametric bias may have encoded not only speaker-dependent information, but also recording-dependent information. However, from the viewpoint of performance of phoneme- and word-discovery, the experimental results suggested that DSAE-PBHL could subtract such information as well. However, the recording environment and other information (e.g., prosody information) might also affect acoustic features. Considering a variety of additional information and developing a robust phoneme and word discovery system is also our future challenge.

In the current model, DSAE-PBHL and NPB-DAA were separately trained. However, as end-to-end learning in numerous deep learning-based models have indicated, the simultaneous optimization of feature extraction and post-processing is essential. We also intend to study the simultaneous optimization of representation learning and phoneme and word discovery in the future.

## Data Availability Statement

The datasets and source codes used for this study are available in our GitHub repository. Multi-speaker AIOI dataset: https://github.com/EmergentSystemLabStudent/multi_speaker_aioi_dataset; NPB-DAA: https://github.com/EmergentSystemLabStudent/NPB_DAA; DSAE-PBHL: https://github.com/RyoOzaki/DSAE-PBHL.

## Author Contributions

RN developed the method, implemented the original code, performed the experiment, and analyzed the results. RO evaluated the methods and the maintained the software. TT contributed to development of theory and formulation, and wrote the paper.

### Conflict of Interest

The authors declare that the research was conducted in the absence of any commercial or financial relationships that could be construed as a potential conflict of interest.

## Footnotes

^1^It is widely established that infants can distinguish individuals around them in their early developmental stage. Therefore, the assumption is reasonable from the developmental perspective as well.

^2^Hidden Markov Model Toolkit: http://htk.eng.cam.ac.uk/

^3^http://scikit-learn.org/stable/

^4^Julius: http://julius.sourceforge.jp/
